# A Smart Robotic Walker With Intelligent Close-Proximity Interaction Capabilities for Elderly Mobility Safety

**DOI:** 10.3389/fnbot.2020.575889

**Published:** 2020-10-22

**Authors:** Xiaoyang Zhao, Zhi Zhu, Mingshan Liu, Chongyu Zhao, Yafei Zhao, Jia Pan, Zheng Wang, Chuan Wu

**Affiliations:** ^1^Department of Computer Science, Faculty of Engineering, The University of Hong Kong, Hong Kong, Hong Kong; ^2^Department of Mechanical Engineering, Faculty of Engineering, The University of Hong Kong, Hong Kong, Hong Kong; ^3^Department of Mechanical and Energy Engineering, Southern University of Science and Technology, Shenzhen, China

**Keywords:** elderly safety, human-robot interaction, intelligent control, falling protection, soft-robotic interface, coaxial front following, sound source localization

## Abstract

The elderly population has rapidly increased in past years, bringing huge demands for elderly serving devices, especially for those with mobility impairment. Present assistant walkers designed for elderly users are primitive with limited user interactivity and intelligence. We propose a novel smart robotic walker that targets a convenient-to-use indoor walking aid for the elderly. The walker supports multiple modes of interactions through voice, gait or haptic touch, and allows intelligent control via learning-based methods to achieve mobility safety. Our design enables a flexible, initiative and reliable walker due to the following: (1) we take a hybrid approach by combining the conventional mobile robotic platform with the existing rollator design, to achieve a novel robotic system that fulfills expected functionalities; (2) our walker tracks users in front by detecting lower limb gait, while providing close-proximity walking safety support; (3) our walker can detect human intentions and predict emergency events, e.g., falling, by monitoring force pressure on a specially designed soft-robotic interface on the handle; (4) our walker performs reinforcement learning-based sound source localization to locate and navigate to the user based on his/her voice signals. Experiment results demonstrate the sturdy mechanical structure, the reliability of multiple novel interactions, and the efficiency of the intelligent control algorithms implemented. The demonstration video is available at: https://sites.google.com/view/smart-walker-hku.

## 1. Introduction

Over the last few decades, the elderly population has rapidly increased globally and is expected to exceed 2 billion by 2050 (WHO, 2018). While constrained physical and cognitive abilities leave many older adults dependent, most of them prefer to continue living in their homes rather than moving into nursing homes, because the opportunity to stay in a familiar home environment offers them greater privacy and autonomy (Garçon et al., 2016).

For patients with Parkinson's disease, movement disorder can severely disrupt the performance of daily activities and increase the risk of falling. Despite various existing walkers are owned by seniors, reported statistics show that 33% of people over 60 years fell at least once (Luz et al., 2017). We argue that intelligence is essential for an elderly walker to detect abnormal user behaviors and provide timely safety support, since primitive assistance devices, such as rollators and walkers, are much likely to fail (Bertrand et al., 2017). Exoskeleton (Tucker et al., 2019) is another approach with multiple robotic joints and links worn onto the user body, effective but less practical for daily wearing by older persons. Moreover, rather than merely using remote button (Glover, 2003), voice (Gharieb, 2006), or gesture (Gleeson et al., 2013) to achieve user interaction, older persons need various modes of human-robot interaction for convenience and efficiency. These motivate us to seek a solution of equipping the robotic walker with sufficient intelligence and interaction to guarantee mobility safety of older users.

In this paper, we propose a novel smart robotic walker that targets a convenient-to-use indoor walking safety aid for the elderly. Present-day assistant devices require attentive control of the user while moving (Di et al., 2015; Xu et al., 2018), which could raise safety issues for many elderly people with executive dysfunction or dementia. Although a few studies have investigated the task enabling the walker to follow behind the user (Moustris and Tzafestas, 2016), the problem is simplified since the human intention is known a posteriori by inspection of his/her trajectory. We take the approach of adopting a co-axial differential drive with sufficient braking force and enabling our walker to monitor and predict the movement trend of the user by detecting gait posture, our walker can then automatically move in front of the user, providing mobility support. With the walker moving in the front, we can enforce the elderly walking in a forward-learning position, preventing retropulsion falls, while our walker can support propulsion falls; with auto moving functionality, our walker alleviates the older users from attentive control of the walker.

As a service robot for the older users, the user interface (UI) provides the fundamental information acquisition for any intelligence and human-robot interaction. The vast majority of existing service robots often choose a touch-screen panel for touch input (Hans et al., 2002; Graf, 2009). However, there are severe limitations for touch-screen UI, from not being able to provide user motion data, to only detecting user command with a pre-defined set of items. Given that soft robotics has become a new trend to design and fabricate robots from a very distinctive approach than conventional robotics (Yi et al., 2018), we propose to use a soft robotic layer to be the user interface in constructing the handles due to its inherent safety (lack of rigid components) (Chen et al., 2018) and intelligence add-ons. To measure user intention and detect emergency event (e.g., falling) in a timely manner, we embed a sensor network inside the soft chamber to monitor force pressure on the handles. After conditioning and asynchronous filtering of the pressure data, our walker generates the appropriate output for system execution to meet user demand or provide safety support.

We also consider the very likely scenario that the elderly user and the walker are located in different locations in a household (e.g., the walker being charged and the user in bed). The autonomous mobility of an elderly walker through user voice summoning becomes essential to provide ready assistance to users with mobility impairment, which is often neglected in existing design (Mukai et al., 2010; Xu et al., 2018). To localize the sound source for autonomous mobility, existing methods have used Time Difference of Arrival (TDOA) (Valin et al., 2003) or deep neural network (DNN) (Ma et al., 2018), which are often ineffective in long distance or a multi-room environment. Recently, reinforcement learning (RL) has been widely applied in robotics. The mobility system based on RL (Zhang et al., 2015), for the first time, learns robotic manipulator motion control solely based on visual perception. Tai et al. (2017) learn to navigate by training a policy end-to-end, but the solution is only validated on a robotic platform with low degrees of freedom. A novel DRL approach (Choi et al., 2019) with LSTM embedded is proposed to learn efficient navigation in a complex environment. For multi-robot motion control, a decentralized RL model is presented to learn a sensor-level collision avoidance policy in multi-robot systems (Fan et al., 2020). Domains like UAVs (Hu et al., 2020; Wan et al., 2020) and underwater vehicles (Carlucho et al., 2018; Chu et al., 2020) have also exploited RL for motion control for various purposes, e.g., robust flying, path planning and remote surveillance. In our work, we present a novel approach of exploiting mobility of the walker and RL techniques for efficient sound source localization (SSL).

We conduct extensive experiments to demonstrate the efficiency of our smart walker for elderly mobility safety in the following aspects:

1. A sturdy mechanical structure that fulfills expected functionalities and supports a user of average weight in home scenarios or outdoor sites with slopes ≤ 16°, which outperforms the safety requirement of the related ISO standard (ISO, 2003).
2. Ability to track the user in the front to provide close-proximity walking safety support and turn according to user's turning intention with small error, through detecting lower limb gait of the user.
3. Soft robotic user interface with a finite-state machine (FSM) model to detect user intention and emergency event effectively, ensuring timely safety protection.
4. Autonomous mobility through RL to locate the user (sound source) and navigate to the user, in a multi-room household with environmental noises, reverberations, and long distance (over 10 m).

## 2. Materials and Methods

### 2.1. System Overview

**Figure 1:**
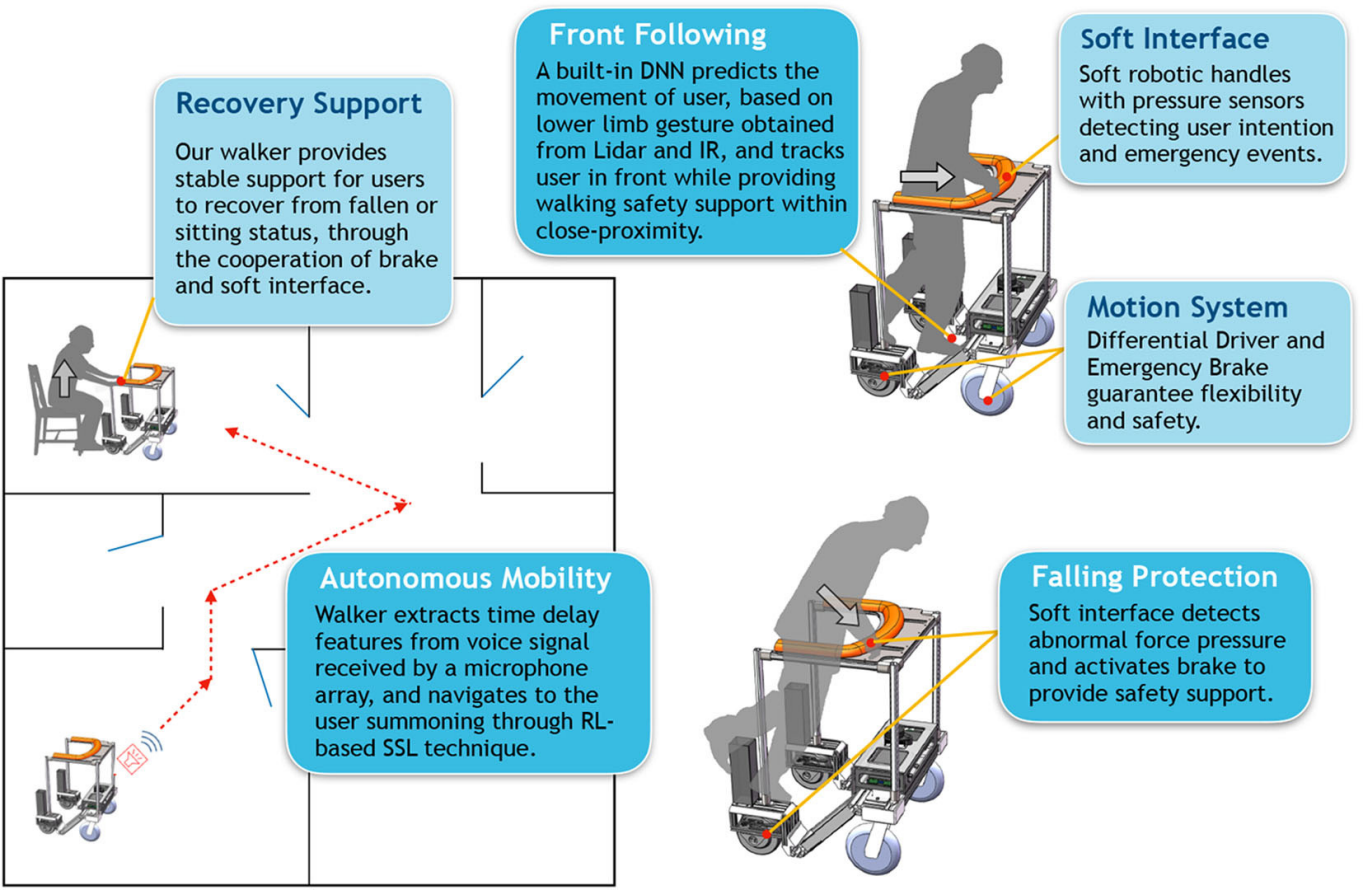
System overview of the smart robotic walker.

An overview of the proposed smart robotic walker with novel functionalities is shown in Figure 1. Our walker consists of a sturdy body frame with sensors deployed at appropriate positions, a motion system with differential driver and emergency brake, and a soft robotic interface with haptic monitor. A user staying in a different room from where the walker is can summon it to come close with the help of RL-based SSL technique, and the brake will be activated to prevent slipping once user intention of entering the front-following status (AKA user walking stage) is detected by the soft interface; then the walker enters front-following walking-assistance status, when a DNN-based method predicts movement of the user to achieve smooth front following with close-proximity safety protection. The force pressure applied on the soft interface is always monitored and analyzed. If an emergency event is detected (e.g., falling), the brake will be activated, and the sturdy body frame and the soft interface will serve as a safety support.

In this section, we will briefly describe our objectives of hardware design and the technique roadmap of software control in order to achieve the proposed functionalities.

#### 2.1.1. Hardware: Smart Walker With Soft Sensing Handle

The hardware design of our smart walker takes cues from both the conventional mobile robotic platform and traditional rollator. Several requirements are met in terms of structural stability, human-robot formation and human-robot interaction.

##### 2.1.1.1. Structural stability

Designing a mechanical structure that is sturdy, strong, and agile enough to provide the safety support for the human user, is a fundamental requirement of the walker. Loading capacity should be sufficient to withstand a human user of ≤ 85 kg leaning against the top handle, with minimum tipping or sliding. To allow the device suitable for home usage, the maximum width of the walker should be no more than 700 mm, to ensure agility when navigating through narrow places.

##### 2.1.1.2. Mobility system

Proposed functionalities require the walker to freely turn into any direction at any time. The walker should achieve zero turning radius or small radius turning in order to navigate within confined spaces. The standard solutions to omni-turning, i.e., omni-wheels or *Mecanum* wheels, have very limited rigidity against tipping disturbances. Besides, holonomic drive is not required as elders rarely walk sideways. In this work, to combine multi-terrain adaptability and standing support, a differential drive is ideal as it is the most widely used mechanism for moving robots and the most effective in terms of control strategy. To further increase safety, a brake mechanism that can respond to emergency in a timely manner is implemented as well.

##### 2.1.1.3. Sensing network

The platform is equipped with a sensing network that enables the walker to perceive and interact with users. Sensing the status of the walker and the user are crucial for intelligent control to ensure user's safety and maximize system performance. This requires equipped sensors to achieve precision, timeliness, and robustness when dealing with various situations. We use optical, thermal, force, and vocal sensors to create a multi-modal sensing network, achieving effective human-robot interaction.

As the most direct way of haptic interaction, we adopt a novel soft-robotic technology (Chowdhary et al., 2019) to construct user interface on the handles for better and safer interacting experience comparing to the existing products.The handles are designed to be soft with certain elasticity to withstand falling shock and provide comfortable touch. With the physical data (force, pressure, etc.) collected by high-sensitivity sensors inside the soft chamber, the system can acquire some useful information about the user all the time.

#### 2.1.2. Software: Intelligent Control

We adopt learning-based methods to achieve intelligent control of the walker, based on signals obtained during human-robot interactions.

##### 2.1.2.1. Soft haptic monitor

The soft interface on handles can detect user's intention and status. With pressure data collected from embedded sensors, we design a finite-state machine (FSM) model to analyze the temporal and spatial characteristics of the pressure data. Based on these characteristics, the intelligent framework is able to infer current status of the user and produce corresponding system actions to support the user in case of potential emergency (e.g., teetering, falling). The touch history of the handles can also be recorded as less privacy-sensitive health data for healthcare personnel to inspect.

##### 2.1.2.2. Close-proximity coaxial front following

To achieve user tracking from the front, the gait information is collected by an infrared temperature sensor and a lidar sensor. We train a neural network (NN) model to learn the intention of the user from time-serial gait data. After obtaining the intention of the user, we compute a target position of the walker to ensure that one foot of the user is on the rear-wheel axis of the walker and the forward direction of the walker is parallel with the orientation of the foot. Such close-proximity and coaxiality between the walker and the user provide timely protection when the user is walking with the walker.

##### 2.1.2.3. RL-based SSL

To achieve autonomous mobility in case that the walker and the user are located at different places (e.g., two rooms in a household), voice signals are monitored by deployed microphone array in a low-power state. Time-delay features of each microphone pair are extracted to estimate the direction of the sound source, and then the walker can move toward it, when the user summons the walker to come close with certain keywords. Before usage, we first train a NN model using supervised learning, on dataset collected from GSound simulator (Schissler and Manocha, 2011). Trained NN model is then fine-tuned through online RL, in daily usage of the walker. Note that during autonomous movement of the walker, once haptic touch is detected by the soft interface (i.e., when the walker reaches the user), the walker can provide sturdy support for the user to recover from sitting or lying status (if he/she has fallen on the floor).

### 2.2. Hardware Design

**Figure 2:**
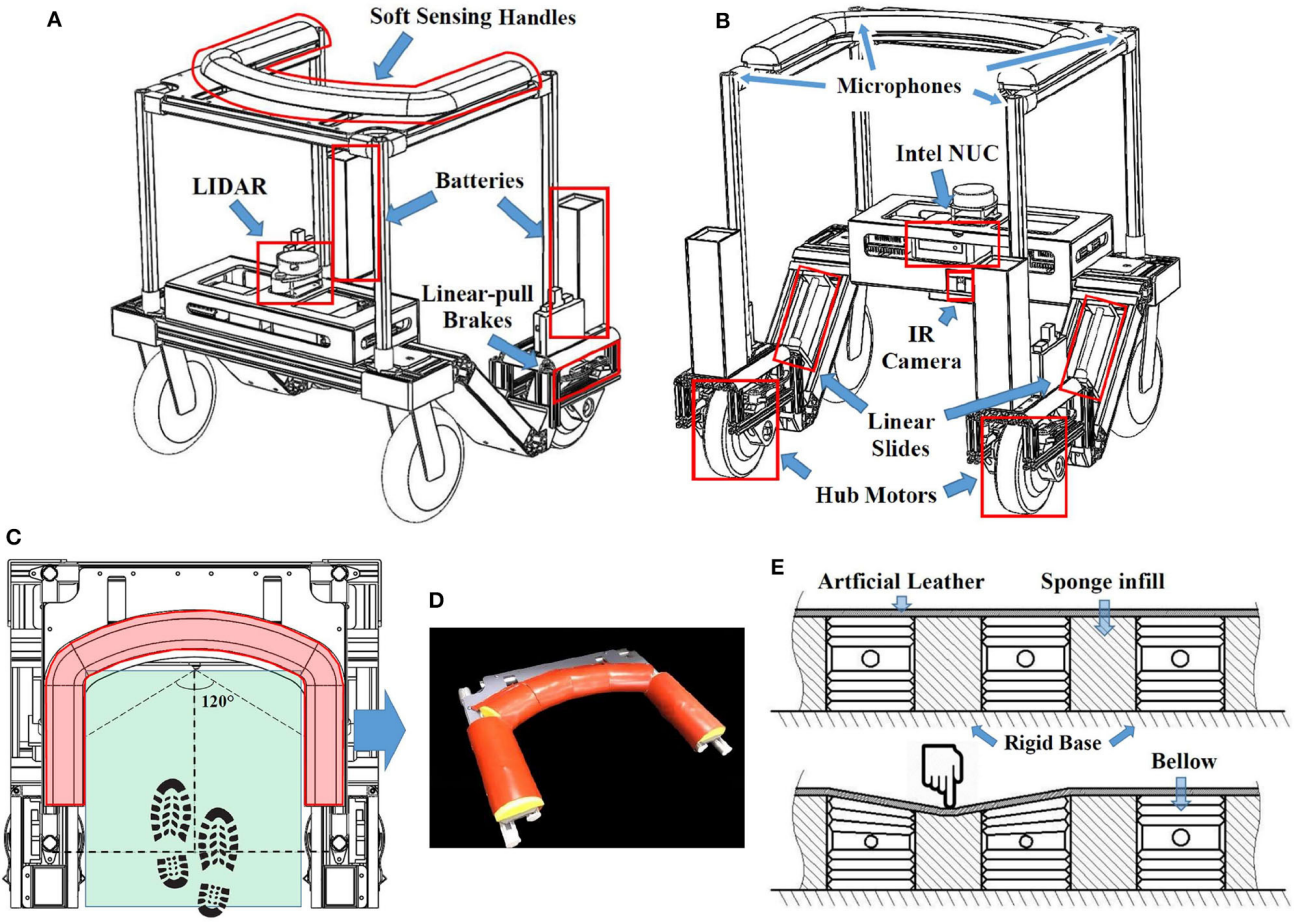
The overview of the smart walker: **(A)** Front view; **(B)** Rear view; **(C)** Top view; **(D)** Soft sensing handle; **(E)** Section view of the handle.

Hardware structure of the proposed smart robotic walker prototype is shown in Figure 2, consisting of the chassis and the upper handle, with basic parameters given in Table 1.

**Table 1:** Basic parameters of the smart walker.

**Parameter**	**Value**
Width * Length * Height	660 mm * 626 mm * 832 mm
Weight	37 kg
Minimum Turning Radius	622 mm
Speed	0–5.34 m/s

#### 2.2.1. Body Frame and Actuation

The design of the body frame takes many safety issues into consideration. For static and dynamic stability, weight is concentrated low into the chassis. The resulting center of gravity (CG) height *H*_*CG*_ as estimated in Solidworks (Dassault Systems S.A.) is 156 mm, i.e., 18.8% of the walker's total height. The maximum tilt angle Φ_*max*_ before the CG goes over the supporting point (assuming the CG is located at the center point in the lateral direction) can then be calculated as:

$$\begin{array}{l} \Phi_{max}=arctan\frac{l/2}{H_{CG}} \end{array}$$

where *l* = 540 mm is the length of wheel base. Calculated Φ_*max*_ is approximately 60°, which is significantly larger than the tilt angle that human can incur before losing balance. Thus, before the tilt angle of walker reaches Φ_*max*_, walker's weight (37 kg) would help elderly users resume to an upright position.

The walker protects the user from his/her front and both lateral sides to prevent falling, forming a “C” shape from the top view (Figure 2C). To maximize the space for the user to walk within the range of support (green area in Figure 2C) while constraining the maximum length of the walker, two wheel-hub motors (*Zhongling Technology* Ltd., China) are used as the rear driving wheels due to their lateral compactness. This results in providing a 420 × 436 mm walking space with the maximum walker width of 660 mm.

Both wheels are equipped with individual emergency brakes, modified from bike brakes, actuated by two individual linear actuators with 0.4 s of lead time to maximum break force, ensuring fast emergency response (Ahn et al., 2019).

#### 2.2.2. Sensor Arrangement

To measure the movement of the walker, each wheel is equipped with a high-precision rotary encoder (4,096 ticks per revolution) and a wheel encoder odometer is implemented as well. The odometer yields the position of the walker (*x, y*, δ)_*walker*_, where (*x, y*)_*walker*_ indicates the position and δ_*walker*_ indicates the orientation of the walker in the global frame, and the moving state (*v*, ω)_*walker*_, where *v* is the linear velocity and ω is the angular velocity of the walker, over time. An inertial measurement unit (IMU) is used to correct the orientation as it uses magnetometer to measure the yaw movement, which is more robust to dynamic disturbance as compared to the odometer.

Multiple sensors are used to acquire user states in order to achieve functions such as hand-free front following and SSL. The lidar used for leg detection is placed lower than the user's knees to ensure a good leg separation result. It is mounted in the front at a height of 410 mm, which is about the height of the upper calf of humans. An IR thermometer which has a 120° field view is placed at an angle that can cover most of the walking area and the user's front foot when walking. Four microphones are installed at the top of the walker with fixed spacing between each other. This will lead to different input signals at the microphones received from the same sound source, helping the controller to locate the source. All the readings from various sensors will be sent to a small form factor PC (*NUC, Intel* Co.) for processing.

#### 2.2.3. Soft Sensing Handle

We use a soft robotic layer to be the user interface when constructing the handles (Figure 2D) as a safe and friendly approach of interaction. In the core of a handle, the rigid base made of acrylic board is used to transfer the load to the main frame. The interior of the handle consists of multiple air pressure sensing bellows connected with air pressure sensors (Figure 2E), and sponge infills to provide a consistent handle surface. The pressure sensors detect the normal air pressure inside the bellow when the user is not holding the handle. When the user grabs onto the handle, a sudden change in air pressure will be read almost instantly through an MCU (Arduino Mega 2560). By deciphering the air pressure signal, the system calculates whether the user is or is not holding, or how firm the grip is. The handle is covered with a layer of artificial leather providing comfortable texture.

From each pressure bellow we can extract the information of pressure changes and the rate of changes. To enrich the sensing capability of the handle, the slight rigidity of the covering leather acts as a linkage between separated bellows. In this case, even when a force is not directly exerted on a bellow, it will also cause a less significant pressure change in the adjacent bellows (Figure 2E). Hence, one more dimension of signal, which is the position of the exerted force, is added to the sensing network. The reliability of this prototype handle will be tested in section 3.

### 2.3. Software Design

We next present the detailed design of software techniques to achieve intelligent control: (i) soft haptic monitor to recognize pressure pattern, (ii) close-proximity front following, and (iii) sound source localization through a reinforcement learning (RL) model.

#### 2.3.1. Soft Haptic Monitor

Pressure data collected by sensors on the soft haptic handle with temporal and spatial characteristics can be analyzed to monitor the state of the user.

##### 2.3.1.1. Position adjustment

**Figure 3:**
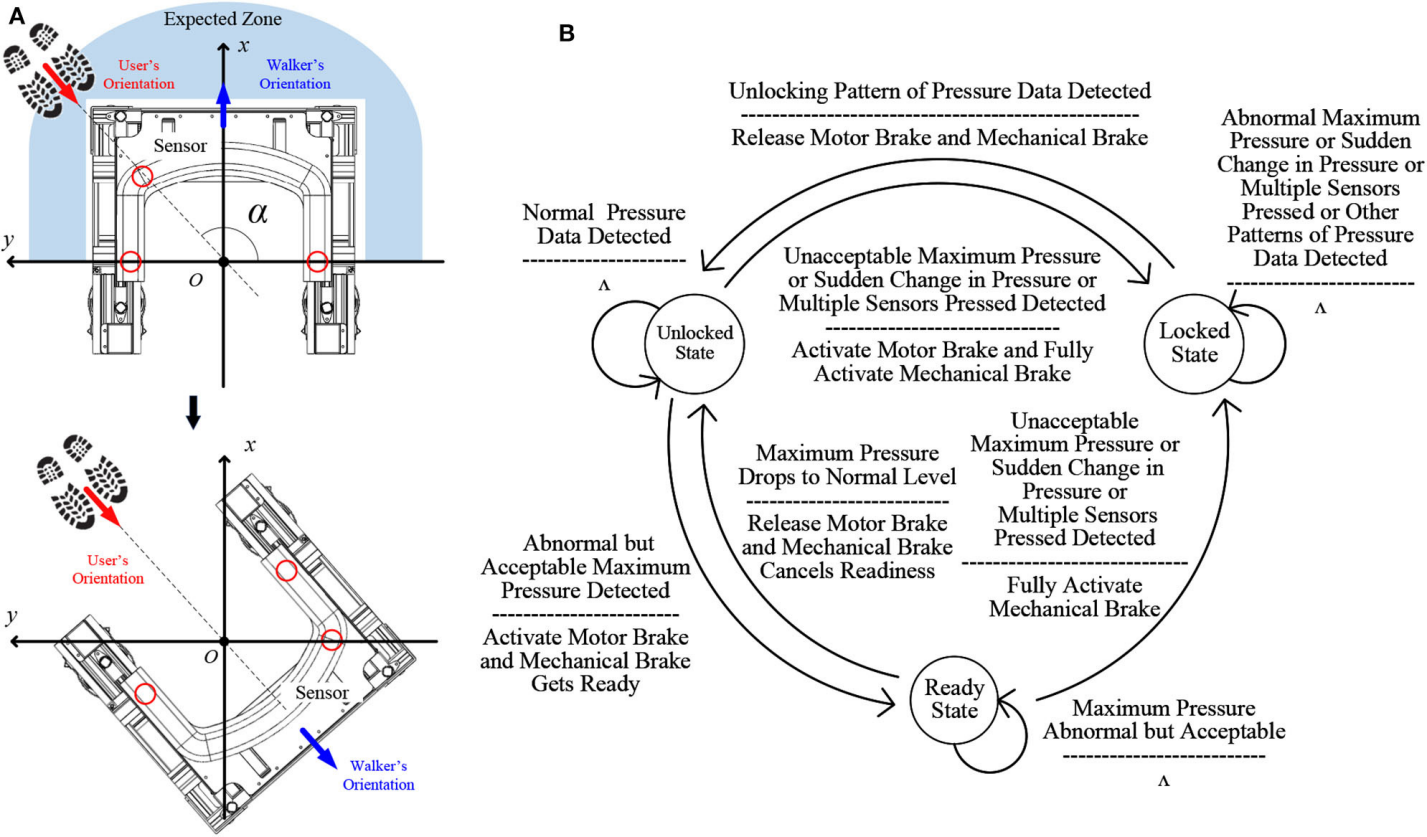
**(A)** Walker position adjustment upon reaching user after SSL; **(B)** FSM of the soft haptic monitor.

After the walker navigates to proximity of the user (through SSL), the walker needs to know how itself is positioned against the user, and adjusts itself to be well-positioned as the user's walking support, before it enters the walking-support status (i.e., starting close-proximity front following of the user). Figure 3A illustrates the relative positioning of the user and the walker when the walker has moved up to the user through SSL. The user is likely to be in the Expected Zone because the walker keeps heading to the sound source. Even if the user is not in the Expected Zone, he/she can call the walker through voice control and the walker will adjust its direction again and eventually the user will be in the Expected Zone. The user can press his/her nearest part on the soft haptic handle to let the walker know where he/she is. Taking the midpoint of the line connecting the two sensors at the two ends of the handle as the origin *O* and the connection line as the *y* axis, a rectangular coordinate system is established. The connection line between the origin *O* and the center of a sensor has an angle α with the *y* axis. According to α, the walker will rotate at a calculated angle of β so that the walker and the user will be facing the same direction. The rotation angle β and the rotation direction are calculated as follows:

$$\begin{array}{l} \beta =\{\begin{array}{cc} \frac{\pi }{2}+\alpha \;right, & 0<\alpha <\frac{\pi }{2},\\ \frac{3\pi }{2}-\alpha \;left, & \frac{\pi }{2}\leq \alpha <\pi . \end{array} \end{array}$$

##### 2.3.1.2. State monitor and falling protection

When the user is operating the walker, the soft haptic handle will monitor the state of the user. Different states are related to different intentions of the user. As an interface, the soft haptic handle collects the pressure data to infer the states of the user. For example, when there is a fall, there will be an abnormally high pressure or a rapid pressure change. Also, multiple sensors may be pressed since the user tends to lean on the handle when he/she falls. To detect user's intention and falling, the pressure data from different sensors of the soft haptic handle are analyzed independently or collectively: independent analysis concerns the pressure on one specific sensor, while the collective analysis focuses on comparing the pressure and changes of pressure on different sensors.

1. **Abnormal Maximum Pressure:** One case of independent sensor analysis is that we calculate the maximum pressure value *P*_*max*_ of all pressure values of all sensors at the same time. According to *P*_*max*_, the motor brake and the mechanical brake will be activated differently. The former can be activated instantly but is not sturdy enough to support the user, while the latter needs time to be fully activated but can provide more satisfying braking force. Especially, if *P*_*max*_ is normal (e.g., *P*_*max*_ is below the pressure generated by the user when he/she is grabbing the handle but is not leaning on it), the user is in a safe situation and the walker operates normally. When *P*_*max*_ becomes higher but still in an acceptable range, the user is at the boundary state between a normal situation and an accident, e.g., the case when the user stops and leans a little on the walker for a rest. In this range the user can recover to safe situation easily just by straighten his/her body without pushing the walker hard. In such a situation, the motor brake will be activated and the mechanical brake will change to a readiness state. The threshold that differentiates the normal range and the acceptable range is about 5% of user weight. If the value of *P*_*max*_ falls and becomes in the normal range again, the motor brake will be released and the mechanical brake will cancel the readiness state; the walker can move again. On the other hand, if *P*_*max*_ keeps raising and becomes unacceptable, representing the user leans more heavily on the walker, the mechanical brake will be fully activated and the walker will be locked to offer stable support for the user; the walker will not be unlocked until the soft haptic handle identifies an unlock intention from the user (to be detailed later). The threshold that differentiates the acceptable range and the unacceptable range is about 8.5% of user weight. The two thresholds can be changed by the user within 2% of his/her weight for better user experience. For privacy concern, user can choose one of the preset levels of weight range for threshold calculation. The preset levels consist of low weight (e.g., 40–55 kg), medium weight (e.g., 55–70 kg), and high weight (e.g., 70–85 kg). We use the average weight of each level range to decide the thresholds.
2. **Abnormal Pressure Change:** Another independent analysis situation focuses on the maximum change rate of $R_{max}^{\prime }$ of all pressure values of all sensors. For each sensor, the current pressure value is used to minus the previous pressure value. Then the difference is divided by the time in between, about three sampling periods, to get all the change rates of all the sensors. Among all these change rates, the maximum change rate $R_{max}^{\prime }$ is calculated. With $R_{max}^{\prime }$, the walker can detect an accident and offer protection sooner. For example, in cases of a stumble or a fall when the user is walking, the change rate $R_{max}^{\prime }$ is very large (regardless of *P*_*max*_'s value); this is considered as an accident and both two brakes will be activated immediately, and the walker will be locked. The threshold for detecting the accident is about 15% of user weight per second. The sudden change of pressure can be detected within 0.2 s.
3. **Multiple Sensors Simultaneously Pressed:** One collective analysis situation is that when too many sensors are being pressed at the same time, a falling tendency can be detected. Among the pressed sensors, if there are only sensors from the left and right sides of the soft handle, the user is inferred to be holding the two sides; if there are sensors from the front part of the soft haptic handle triggered, the user is assumed to lean on the front part of the handle and need support from the walker. In this situation, both brakes of the walker should be fully activated and the walker should be locked.
4. **Pressure Change Comparison:** Another collective analysis situation focuses on temporal characteristics of pressed sensors. By comparing different pressure changes on different sensors, we can detect the strength and direction of the force applied by the user to the soft haptic handle. There are different cases: (a) When the two brakes are fully activated, if the user grabs the handle for recovery from fallen or sitting status, the direction and the strength of the force applied on the handle changes over time. If the change rates and the time for pressure value reaching the peak of different sensors are different or *P*_*max*_ is very high, we infer that the user needs support, and the two brakes will not be released. (b) If the user gently puts his/her hands on the left and right sides of the handle, the pressure data collected from different sensors on the two sides vary a bit over time, while the change rates and the time for reaching the peak will be similar among the sensors because the direction of the force remains unchanged and *P*_*max*_ is also at an acceptable level. We regard these characteristics as a signal of unlocking, and the walker will be unlocked and the two brakes will be released. (c) When the handle detects that the pressure of the left side is a bit higher than that on the right side, the user may want the walker to turn to the left. We will use such a pattern to decide turning radius of the walker.

By analyzing these pressure data patterns, falling and other user intentions can be recognized. Therefore, the walker can monitor the state of the user and provide falling protection, or respond to other user intentions.

##### 2.3.1.3. The FSM of the soft haptic monitor

An FSM is embedded to control the working of the walker based on user states, as shown in Figure 3B. There are three states: *unlocked state, locked state*, and *ready state*. When the walker is in the *unlocked state*, the motor brake and the mechanical brake are released, and the walker is movable. When the Soft Haptic Monitor detects an accident, such as in cases of an unacceptable large maximum pressure *P*_*max*_, a sudden change in the pressure, or multiple sensors are being pressed at the same time, the motor brake and the mechanical brake will be fully activated immediately and the walker will be in the *locked state*. At this state, the walker will be stable enough to offer support for the user, and will not respond to other signals except the unlocking pressure pattern. The other signals include not only those that activate the conversion from *unlocked state* to *locked state*, but also the patterns that cannot be analyzed as the unlocking pressure pattern such as the pressure pattern of recovery from falling. When the unlocking pattern is detected, the walker's state changes from the locked state to the unlocked state, when the two brakes will be released. When the walker is in the unlocked state, if an abnormal but acceptable *P*_*max*_ is detected, the walker will be in the *ready state*: at this state, the motor brake will be activated so the walker can not move; the mechanical brake will be ready for further protection. If *P*_*max*_ drops back to a normal value, the walker will go back to the unlocked state; if *P*_*max*_ keeps rising and finally becomes unacceptable, the walker will enter the locked state. Since the mechanical brake is ready, it will take less time for the mechanical brake to be fully activated. Accidents like a sudden change in pressure and multiple sensors pressed will also activate this conversion.

##### 2.3.1.4. Speed control

As the interface of the walker, the soft haptic handle allows the user to control the speed of the walker for effective walking assistance. Five speed-levels are preset and the user can select their preferred one by pressing two sensors on the handle. The two sensors are the sensors at the end of the left and right sides of the handle. One is used for acceleration and the other is used for deceleration. For the safety of the user, if one button is pressed, the walker will not respond to the other sensor, i.e., the walker will only respond to one button at a time. If one button is pressed and not released, the speed level of the walker will not keep changing. When the user presses the speed control button, there will be a unique peak of pressure value on that sensor while the pressure on other sensors will be weak. Therefore, this pattern of pressure data is different from other patterns and can be used while the FSM is monitoring the state of the user.

#### 2.3.2. Close-Proximity Front Following

**Figure 4:**
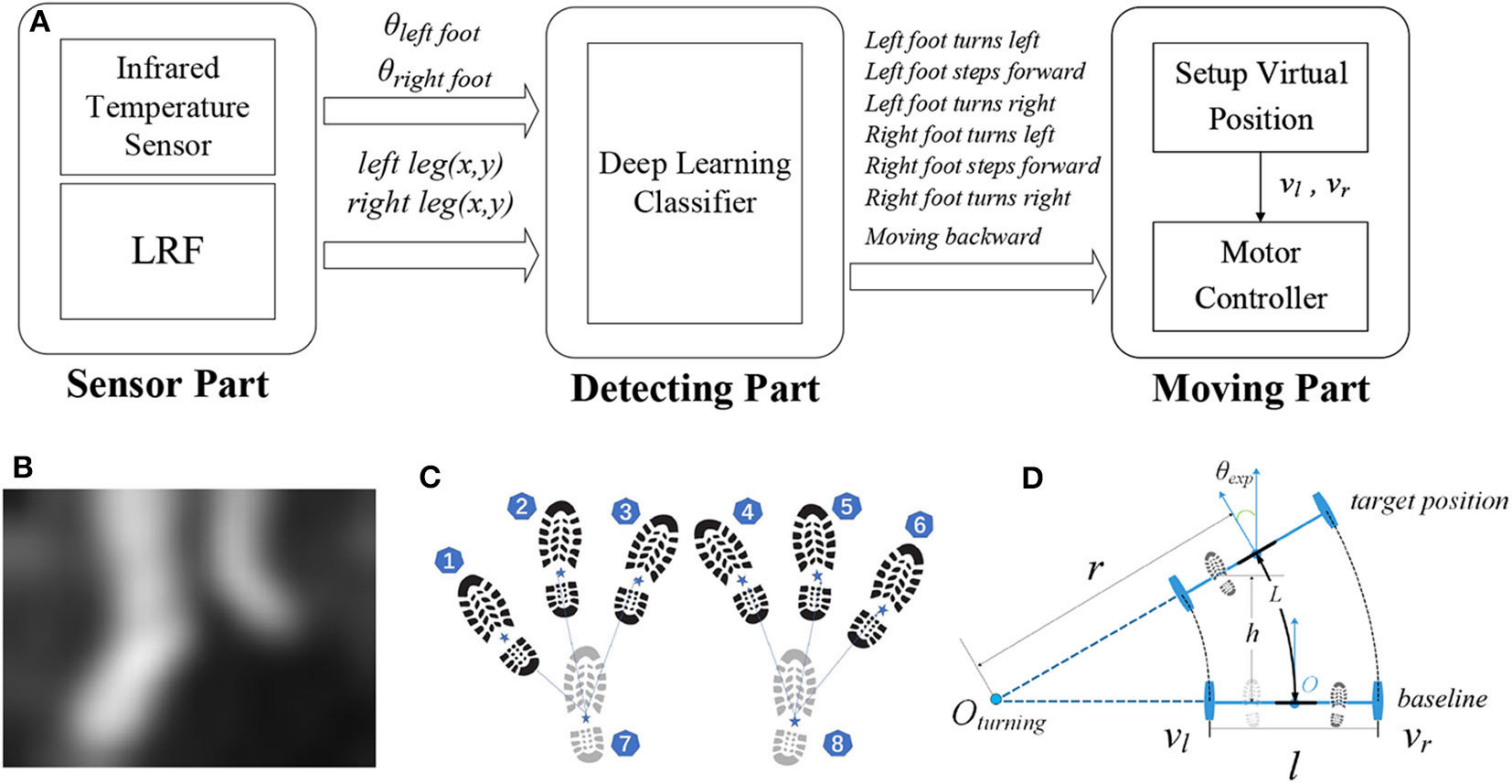
**(A)** Workflow of front following; **(B)** IR image frame; **(C)** gait samples with labels; **(D)** movement control of the walker.

The walker tracks the user in the front through an NN-based intention detecting approach and generates movement through building a virtual target position. See Figure 4A for an illustration.

##### 2.3.2.1. Sensor data processing

The IR sensor returns a 32 × 24 thermal image (see Figure 4B as an example), which can be flattened to a temperature vector $\vec{u}$ with dimension of 768. Compared with an RGB camera, a low-resolution IR sensor as a visual sensor is less costly and more privacy preserving. We normalize data in the temperature vector $\vec{u}$ into image data in a vector $\vec{g}$, where *u*_min_ and *u*_max_ are the maximum value and the minimum value in this vector, respectively:

$$\begin{array}{l} g\left[i\right]=\frac{u\left[i\right]-u_{\min}}{u_{\max}-u_{\min}}. \end{array}$$

Meanwhile, we identify the user leg positions in relation to the walker using data from the lidar sensor. We set a *baseline* to be the straight line connecting the two rear wheels of the walker, and the origin as the midpoint of this baseline. The forward direction is the positive x-axis, and the left direction is the positive y-axis. In this way, we define a coordinate system relative to the origin with the right-hand system, and calculate the coordinates of user leg (*x, y*) relative to the walker as follows:

$$\begin{array}{l} \left[\begin{array}{c} x\\ y \end{array}\right]=\left[\begin{array}{cc} \cos\theta_{walker} & -\sin\theta_{walker}\\ \sin\theta_{walker} & \cos\theta_{walker} \end{array}\right]\left[\begin{array}{c} x_{leg}-x_{walker}\\ y_{leg}-y_{walker} \end{array}\right] \end{array}$$

where (*x*_*obj*_, *y*_*obj*_) and θ_*obj*_ describe the coordinates and orientation of user leg or the walker relative to the initial starting position of the walker, respectively.

We distinguish the computed leg positions into two classes using a k-means algorithm (Krishna and Murty, 1999), and use the prior condition that the *y* value of the left foot is more than that of the right foot to tell which class represents the left or right foot. The image of the two feet and the leg positions relative to the walker are output of sensor data processing.

##### 2.3.2.2. Movement intention detection

Front following is essentially replacing the need of user pushing the walker, such that the walker can move automatically according to user's movement intention. We design an NN model to learn the relationship between user gait and user intention using time-serial data.

Each input sample to the NN is a sequence of 8 data points, where each data point contains vector $\vec{g}$ and leg positions (*x*_*leg*_, *y*_*leg*_) computed from the corresponding IR image. Each sample is labeled with the respective movement according to the user's gait, out of 6 cases (as illustrated in Figure 4C):

1. Left foot turns left, with left foot in the front ① and right foot at the back ⑧;
2. Left foot steps forward, with left foot in the front ② and right foot at the back ⑧;
3. Left foot turns right, with left foot in the front ③ and right foot at the back ⑧;
4. Right foot turns left, with left foot at the back ⑦ and right foot in the front ④;
5. Right foot steps forward, with left foot at the back ⑦ and right foot in the front ⑤;
6. Right foot turns right, with left foot at the back ⑦ and right foot in the front ⑥.

In addition, for the straight-backward case, we can easily tell whether the user is moving backward based on the lidar data, and hence it is not included as one output class from the NN. We use an NN consisting of two 512-unit hidden layers with *ReLU* as the activation function. The output is the probability distribution over the above six cases from a *Softmax* function.

##### 2.3.2.3. Walker movement

Based on the leg positions (*x*_*leg*_, *y*_*leg*_) and inferred movement intention from the NN, we then compute a virtual position that the walker should move to, to achieve front following.

We use the turning radius *r* and a forward or backward distance *h* to decide the moving trajectory (arc length *L*) and the target position of the walker, as illustrated in Figure 4D. Figure 4D demonstrates the rear wheels in the origin and in the target position, respectively. The intersection of the extension of the rear wheels is the turning center *O*_*turning*_. The distance from the center of rotation *O*_*turning*_ to the center of the walker *O* is the radius of rotation *r*.

$$\begin{array}{l} L=\frac{\theta_{exp}\cdot \pi \cdot r}{180}\;\theta_{exp}=\arcsin\left(\frac{h}{r}\right) \end{array}$$

*l* is the length of the driving wheel base; we ensure that there is one foot on the baseline and the orientation of the foot is parallel with the forward direction of the walker. There are three cases:

(a) When the NN output is case (1) or (6), the user is making a left or right turn. The range of this turning radius *r* is *l* to 2·*l*, as determined by the probability *p* corresponding to this case: when the probability is large, the turning intention is obvious, and the walker is given a relatively small turning radius *r*; when the probability is relatively small but is still higher than that of the forward case, the user's intention is to move forward with a turn, and the walker will be given a relatively large turning radius *r*. When the turning center corresponding to the turning radius *r* is on the left side of the walk *O*, the turning radius *r* is positive; for symmetry, when the turning center *O*_*turning*_ is on the right side of the center of the walker *O*, turning radius *r* is negative. Therefore, there are two cases of the turning radius:
  $$\begin{array}{l} r=\pm \left(l\cdot p+l\right) \end{array}$$
(b) When the inferred movement is (2), (5), or moving backward, the turning radius *r* is positive infinity, i.e., *r* = +∞.
(c) When the NN output is case (3) or (4), the user is marking a sharp right or left turn. Generally, these two situations occur after case (a) and are to further complete a sharp turning process. Therefore, we set the turning radius to one half of the walker width to provide a maximum rotation space. We have two cases of the turning radius: $r=\pm \frac{l}{2}$.

Due to differential drive control, two velocities *v*_*l*_ and *v*_*r*_ are calculated to control the walker to move to the target position. In practice, a walker is typically assigned with a linear velocity *v*. The two velocities *v*_*l*_ and *v*_*r*_ of each of the rear wheels can be calculated as follows:

$$\begin{array}{l} v_{l}=v-\frac{v\cdot l}{2r}\;v_{r}=v+\frac{v\cdot l}{2r} \end{array}$$

#### 2.3.3. RL-Based SSL

**Figure 5:**
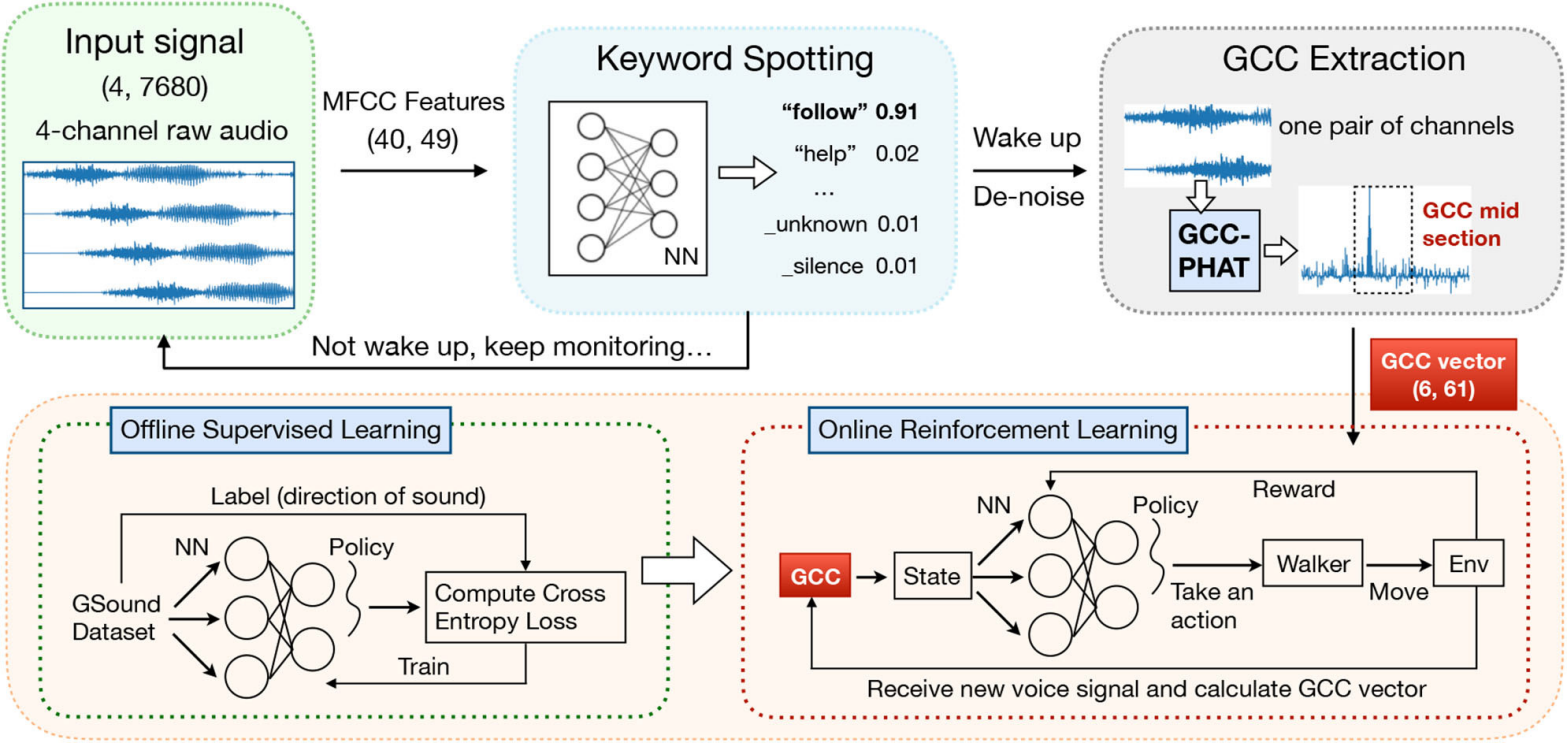
The workflow of sound source localization (SSL) with a series of pre-processing and a reinforcement learning (RL) model.

Our walker monitors audio signal received by a 4-channel microphone array, and can be waked up by customized keywords through a simple keyword spotting system. We choose 1 s of raw audio as input signal. In particular, 40 MFCC features are extracted from a frame of length 40 ms with a stride of 20 ms, which gives 1960 (40 × 49) features for each 1-s audio clip. We use Google speech commands dataset to train an NN model with three hidden layers, each with 128 neurons, to classify the incoming audio clips into one of the predefined words in the dataset, along with the default class “silence” (i.e., no word spoken) and “unknown” (i.e., word not in the dataset). Once waked up, our walker performs SSL following an RL model as follows (see Figure 5 for an overview of the workflow).

##### 2.3.3.1. State space

We define the input state *s* of the RL model to be an *m* × *c* matrix, where *m* is the total number of microphone pairs [e.g., m=(42) if the walker is installed with four microphones], and *c* is the length of the feature vector of one pair. The input state indicates the time difference of arrival (TDOA) of sound signals received at each pair of microphones. The generalized cross correlation (GCC) of two sound signals is a measure of similarity. To accurately calculate the TDOA from the received signals, we firstly perform spectral subtraction (Martin, 1994) to raw audios for the purpose of de-noise and then calculate the GCC-Phase Transform (GCC-PHAT) (Knapp and Carter, 1976) as follows:

$$\begin{array}{l} G_{PHAT}\left(f\right)=\frac{X_{i}\left(f\right){\left[X_{j}\left(f\right)\right]}^{*}}{\left|X_{i}\left(f\right){\left[X_{j}\left(f\right)\right]}^{*}\right|}, \end{array}$$

where *f* is a series of sound data after denoising, *X*_*i*_(*f*) denotes the Fourier transformation of the signal of the *i*th microphone, and []^*^ represents the complex conjugate. Then we compute the *c*-dimensional vector ${\vec{s}}_{n}$ in *s* as the *c*-dimensional subset of *G*_*PHAT*_(*f*), which indicates near-central part of a pair's GCC vector (Knapp and Carter, 1976). We empirically set *c* = 61 to make ${\vec{s}}_{n}$ contain the most useful information of TDOA.

The objective of our SSL module is to output the Direction of Arrival (DOA) of the sound source. Traditional DOA estimation approaches such as the Azimuth Method (Wikipedia contributors, 2020) are often unreliable under high reverberation conditions (as in our scenario) and with complex structures between microphones (as on our walker) (Xiao et al., 2015). We enable the walker to learn the nonlinear mapping from the input GCC features to the DOA output through RL.

##### 2.3.3.2. Action space

After collecting state *s*, the controller selects a horizontal angle (i.e., the DOA) as action *a* based on policy π_γ_(*a*|*s*), which is a probability distribution over action space. The policy is produced by a neural network with γ as the set of parameters. We use a discrete action space including eight angles which are 45° apart: 0°, 45°, 90°, …, 315°.

In output layer of the policy NN, we mask invalid actions, which points to a direction of obstacles within one meter from the walker, by setting their probability to 0 in the probability distribution. Then we re-scale the probabilities of all actions such that the sum still equals 1 (Bao et al., 2019). The walker will then move one meter toward the chosen direction. Note that, while approaching the user, the infrared distance sensors deployed in the front part of the walker will keep feeding distance data (to objects ahead) to the control module. If the walker detects that the user is 5–10 cm in front of it, the walker stops immediately to avoid collision with the user.

##### 2.3.3.3. Reward

We carefully design a reward to use in RL, addressing variability of sound intensity and unknown location of the sound source. Consider a home with one hall and *K* rooms. At the beginning, the walker estimates the user to be in each room or hall with an equal probability, which is the confidence on which room (or hall) the sound source is located in. The walker updates its confidence on each room *k* in every time step:

$$\begin{array}{l} Bel_{t+1}\left(k\right)=\rho \left(z_{t+1},z_{t+1}^{\prime }\right)*Bel_{t}\left(k\right), \end{array}$$

where *z*_*t*+1_ is the vector of relative intensity collected at the microphones during real usage, $z_{t+1}^{\prime }$ is a relative intensity vector of simulated signals when putting sound source at the center of room *k* in our GSound simulator (which emulates the impact of reflection, diffraction and reverberation on sound propagation.), and ρ denotes the Pearson Coefficient. By comparing the similarity of received signals and simulated signals, the walker accumulates probability on each room. Note that after every update, we re-scale *Bel*(*k*)'s to make their sum remains to be 1.

Our reward functions are defined in four cases:

1. When the walker is located in a different room from the sound source, the reward should encourage the walker to step out of the current room *k*:
  $$\begin{array}{l} r_{t}=1-\frac{d_{k}\underset{i\in K\backslash k}{\sum }Bel_{t}\left(i\right)}{Max} \end{array}$$
  where *d*_*j*_ is the shortest distance from the walker to the door of room *k* and *Max* is a constant value for normalization.
2. When the walker is located in the hall, we encourage the walker to explore rooms with higher Bayesian Confidence:
  $$\begin{array}{l} r_{t}=1-\frac{\underset{i\in K}{\sum }d_{i}Bel_{t}\left(i\right)}{Max} \end{array}$$
3. When the walker is located in the same room with the sound source, the difference between direction estimations of consecutive inferences is used as the reward:
  $$\begin{array}{l} r_{t}=1-\frac{\left|a_{t}-a_{t-1}\right|}{315^{\circ }} \end{array}$$
4. When the walker has reached the sound source, it receives a relatively large reward *r*_*t*_ = 5.

##### 2.3.3.4. Offline training and online tuning

The policy NN used by the walker is trained with SGD method (Sutton et al., 2000) by updating the NN parameters γ using policy gradients computed with samples 〈*s, a, r, s*′〉: *a* is the chosen direction for the walker to step forward, *s* and *s*′ are the input state before and after action *a* is taken, and *r* is the reward computed in the current inference.

We collect samples using the GSound simulator (Schissler and Manocha, 2011) for offline training of the NN model, by specifying the locations of a sound source and recording received signals in arbitrary other locations in the multi-room setting. Then we use the trained model in the online setting: during real-world usage of the walker, the NN model is further fine-tuned with collected realistic samples.

## 3. Experiments

In this section, we first conduct experiments to evaluate the mechanical structure of our walker and test sensors deployed on the soft handle. Usability test is also done to prove that our walker can achieve expected functionalities though intelligent control. Specifically, we evaluate walker's ability and efficiency to monitor user intention through soft interface, track user in front within close proximity, navigate to the user based on voice signals. All these demonstrate that our walker is sturdy and agile, with learning-based algorithms implemented to provide elderly users with sufficient mobility safety and effective human-robot interactions.

### 3.1. Mechanical Structure Test

**Figure 6:**
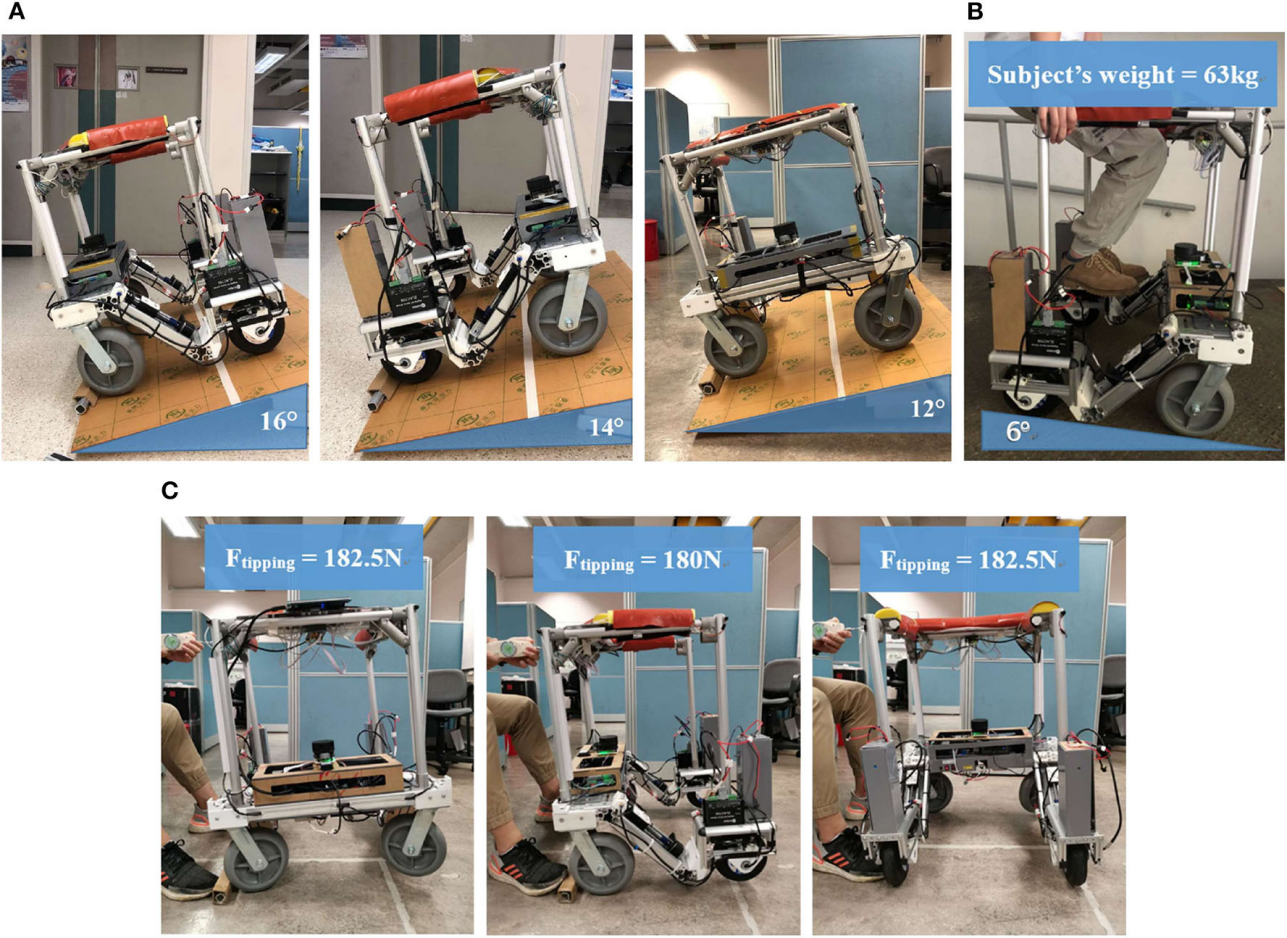
Stability tests; **(A)** Static stability test, from left to right: forward, backward, sideways; **(B)** Dynamic stability; **(C)** Tipping resistance test.

The structural stability is validated according to requirements in ISO (ISO, 2003). For static stability, the ISO standard states that the walker should be placed on a slope in certain ways and it should remain stable without tipping. The slope angle requirement and the corresponding results under different test situations (see Figure 6A) are listed in Table 2. For dynamic stability (see Figure 6B), the self-modified brake mechanism is also reliable as the walker stays stationary when it is placed on a 6° slope with a subject weighing 63 kg leaning on it.

**Table 2:** ISO.11199-2 stability test.

**Test**	**ISO requirement**	**Result**
Forward stability	≥15°	≥16°±1°
Backward stability	≥7°	≥14°±1°
Sideways stability	≥3.5°	≥12°±1°
Brake test	No sliding or 100 mm in not <1 min	No sliding

Moreover, a series of static load tests from the front and lateral directions are conducted (see Figure 6C) to find out how resistant the walker is to the external tipping force. The maximum force exerted on the handle, measured by a spring scale, when the walker starts tipping are 180 N forwardly and 182.5 N laterally. The actual maximum resistance should be larger since the fall rarely happens horizontally.

Overall, our walker passed all the tests with equal or better performance than that required in the ISO standard. It also has good tipping resistance against external push or pull force which plays an important roll in the fall prevention function. Therefore, our structure has strong advantages over those traditional mobility-assistant products, by implementing intelligent autonomous control based on better structural stability.

### 3.2. Soft Handle Evaluation

#### 3.2.1. Sensitivity

**Figure 7:**
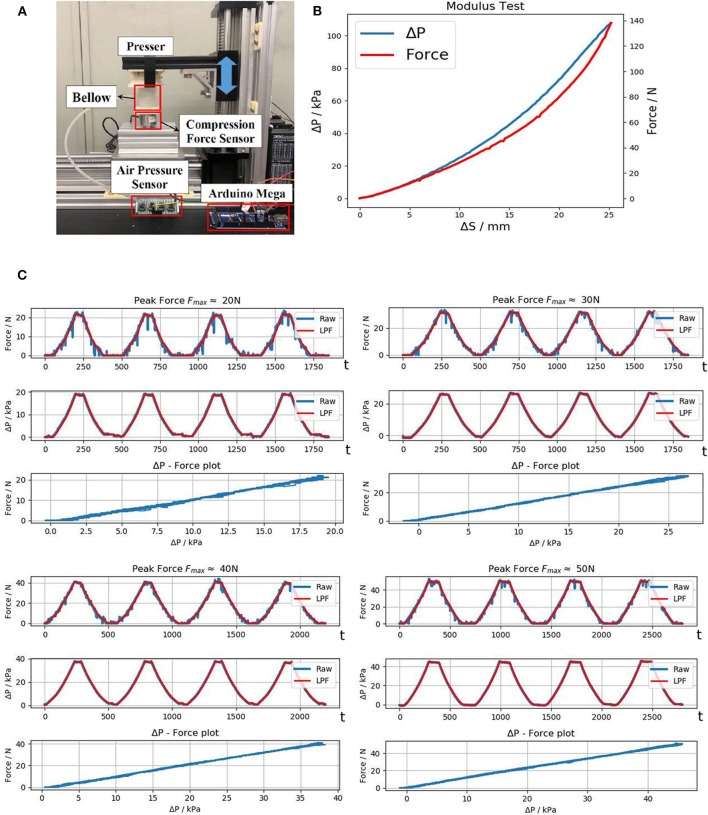
Evaluation of the pressure sensing bellow; **(A)** Testing platform; **(B)** Modulus test; **(C)** Repeatability tests under different external force, from left to right: F_*max*_ ≈ 20N, 30N, 40N, 50N.

To measure the modulus of each pressure sensing bellow and the relation between the load and the pressure detected on it, we implement a dedicated testing platform as shown in Figure 7A. Both pressure change Δ*P* and compression force *F* are measured together and the results are shown in Figure 7B. The maximum load force when the bellow reaches maximum compression (25 mm) is 140 N, giving a modulus of approximately 5.6 N/mm. The normal interaction force in the non-emergency scenarios should be <5 kg. The plot shows that Δ*P* corresponds well with the force exerted on it, laying a good foundation for monitoring user interaction for intelligent control.

#### 3.2.2. Repeatability

The soft handle is expected to have long-term reliable performance and consistency. In the repeatability test, the bellow is gradually pressed until reaching maximum load *F*_*max*_, then it was released to the normal state, and the same process was repeated. The results are passed through a 2nd Order Low-pass Filter (LPF) with a cutoff frequency of 1.50 Hz to remove high frequency noise. The repeatability test plot in Figure 7C shows that Δ*P* and *F* have an almost linear correlation with little deviation throughout the repeated compression. This also indicates that the bellow has good sealing as no negative effect due to air leakage showed up in the test.

### 3.3. Usability Test

We next perform usability tests to evaluate efficiency and practicality of our design in achieving the expected functionalities.

#### 3.3.1. Soft Haptic Monitor

**Figure 8:**
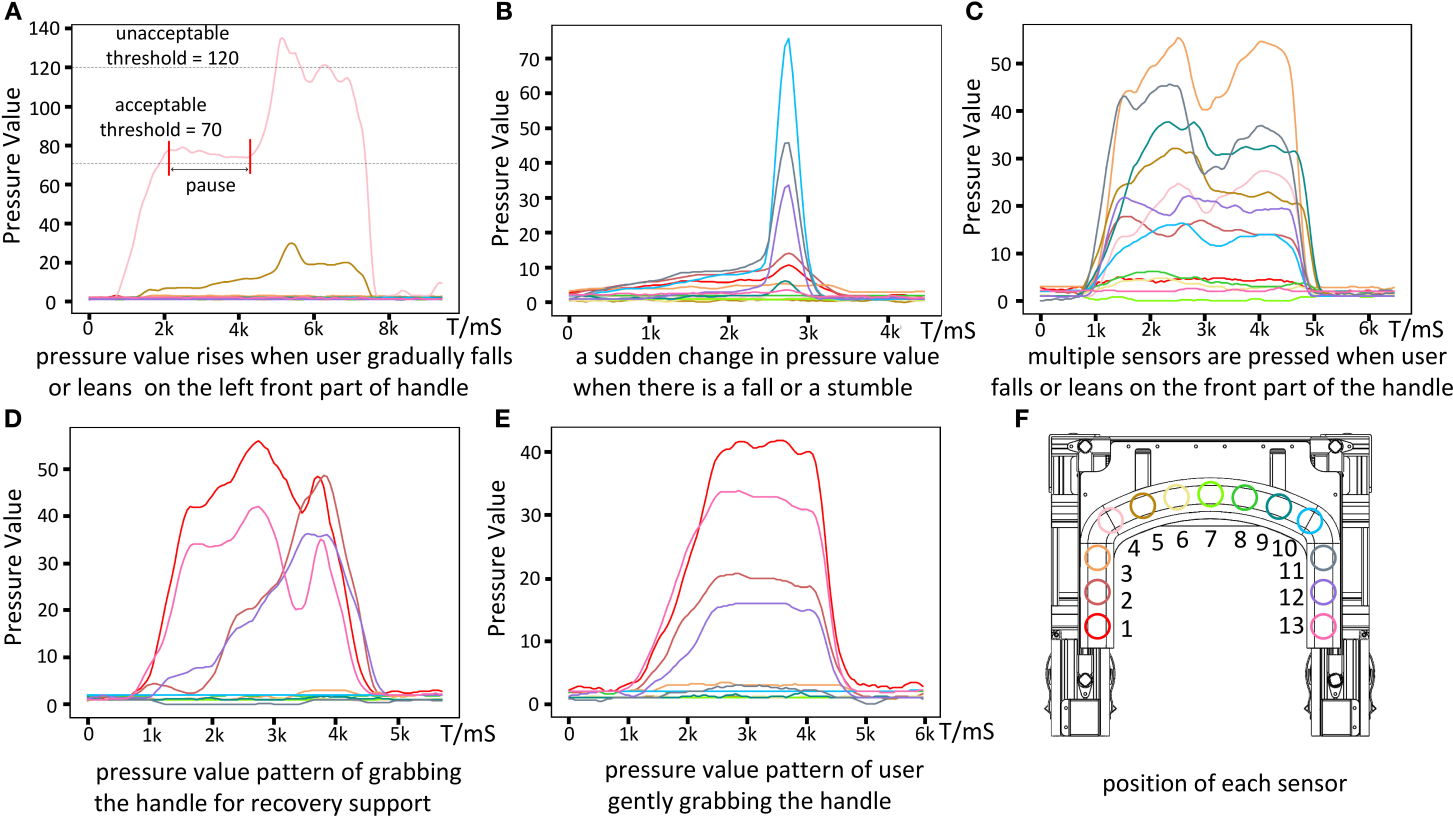
Different patterns of pressure data corresponding to different user interactions and position of each sensor. **(A–C)** Are three possible pressure data patterns when a fall occurs. **(D,E)** Show two types of data patterns when the walker is detecting the user's unlock intention. **(F)** Shows the position of each sensor on the handle.

Experiments are conducted to collect the pressure data of different patterns of touching and grabbing the soft haptic handle. These patterns represent different interactions when the user is operating the walker. The pressure value is transformed from a physical quantity to a raw programmable digital quantity. We record the raw digital quantities of the pressure values from all sensors and their changes over time. A sliding mean filter is applied to pre-process the raw data. The window size of the filter is 5. Figure 8 illustrates the data of different patterns. Figures 8A–C show three possible pressure data patterns when there is a fall or a potential fall. Figures 8D,E illustrate how to detect the unlock intention of the user. Figure 8F shows the position of each sensor on the handle.

Figure 8A shows that when the user is falling, he/she leans more and more onto the left front part of the handle. The maximum pressure *P*_*max*_ of all pressure values at the same time is detected on sensor 4, which rises slowly and eventually exceeds the unacceptable anomaly threshold of the pressure value 120 (e.g., about 8.5% of the user's weight). At the beginning of this process, the walker is in the *unlocked state*. *P*_*max*_ first exceeds the acceptable abnormal threshold of the pressure value 70 (e.g., about 5% of the user's weight), and then the walker goes into the *ready state* for further protection. There is a pause after that, corresponding to the situation where the user is not leaning more onto the handle. Then the user continues leaning more on the walker by putting most of his weight on the handle; *P*_*max*_ keeps increasing and finally reaches the unacceptable abnormal threshold, which brings the walker to the *locked state*.

Figure 8B corresponds to the case of a fall or stumble, with a sudden change in the pressure values. The fastest pressure change rate is about eight times of the change rate of *P*_*max*_ as in Figure 8A, while the peak pressure value in Figure 8B is only half of that in Figure 8A. Such a characteristic can be easily detected and the walker can react promptly to the locked state without waiting for the pressure to rise beyond the anomaly threshold.

Figure 8C shows that when the user falls onto the front part of the handle, multiple sensors (sensors 3–11) are being pressed simultaneously. When the user lies on the front part of the handle for rest and contacts multiple parts of the handle, the pattern of pressure data will be similar. Under these situations, the walker should offer falling protection.

Figures 8D,E show two patterns of user grabbing the handle when the walker is locked and stable. Figure 8D is the case that the user uses the walker for recovery support (from sitting or lying status), when the pressure changes of different sensors and the moments when their pressures reach the peaks are different. The reason is that during recovery, the gesture of the user keeps changing, resulting in the changes of the pressure values and the direction of the force applied to the handle. Figure 8E is the case that the user is ready to walk and gently puts his/her hands on the handle and grabs it. The pressure changes on different sensors and the time of their pressures reaching the peaks are similar. Such a pattern can be used by the user to unlock the walker.

All these results show that the pressure data can be used to detect different states of the user, including falling and other user intention. By analyzing the pressure data, the walker can monitor the user's state to offer falling protection or respond to other user intention. In further development, more applications can be designed to make the walker more intelligent and safer comparing to the current version. For example, by comparing the changes in the pressure over a long period, the walker can detect whether the user is getting tired. Also, more advanced models such as NNs can be applied to learn from the pressure data and extract more information for medical observation.

#### 3.3.2. Front Following

We experiment with user moving forward, turning and moving through narrow space without pushing the handle, as the Supplementary Video 1 demonstrated. For evaluation purpose, we also record actual orientation of the user during front following by having the user wearing an IMU.

**Figure 9:**
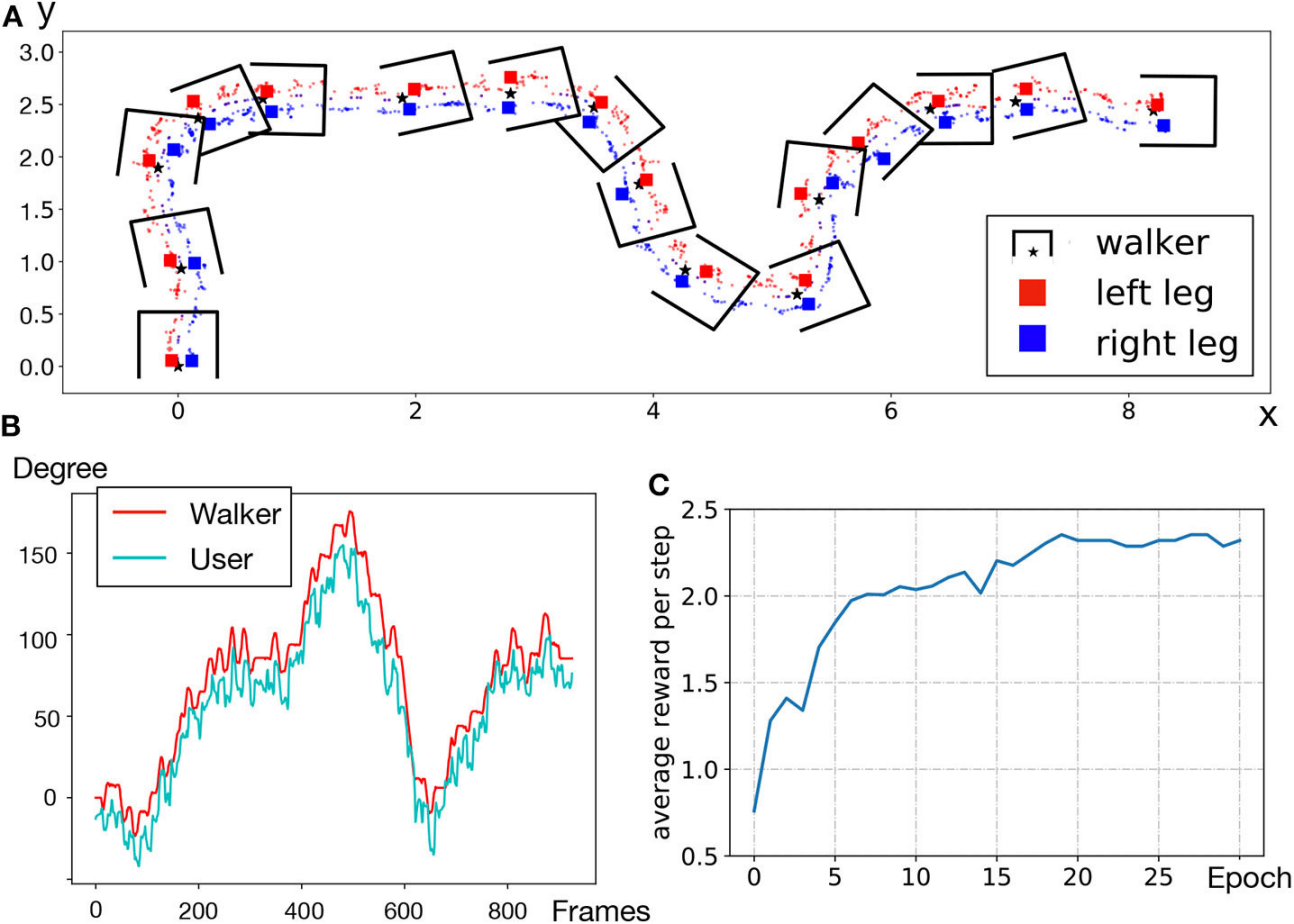
**(A)** Leg position trajectory; **(B)** Orientation of the walker as compared to the user; **(C)** RL training curve of SSL in simulator.

Figure 9A shows user leg positions collected. The x and y axes represent the spatial position, and the unit is meter. Scatter points represent leg locations; we draw a box to represent the walker for every 60 leg points; the center of the walker and corresponding leg positions are highlighted. From the highlighted points, we observe that the walker area always covers both legs and there is always one leg on the baseline. This shows that our front following achieves coaxial following, which is a novel practical function for older persons who cannot consistently push the walker well, but rely on the walker's fall support functionality while walking.

Figure 9B compares the orientation of the user with that of the walker. The range of orientation θ is −180 to 180° and the starting position where the walker enables front following is 0°. We observe that the two lines follow a similar trend, and overlap at some peak points. The average error is 5.5° approximately. It demonstrates that the walker can change direction promptly according to the user's expected angle.

Most existing human-following studies consider robot following the human from the back; in case of robot front-following a human, existing systems assume that the robot is a distance away from the person, and the robot can easily amend its route on the go. In our scenario, the robotic walker and the user are within close proximity (user walking with feet along the rear axis of the walker). While this functionality enables elderly users to achieve hands-free walking, we also consider the situation that elderly users still need physical support with hands touching on soft handles. The intention of turning will then be detected and analyzed through haptic monitor, to cooperate with movement prediction of hands-free front-following to generate better tracking strategy.

The robustness of the proposed NN to predict lower leg gesture can be enhanced in the product usage phase, through collecting diverse and long-term training samples: walkers used by elderly users are allowed to compute and push their model gradients to the cloud periodically, and pull updated model parameters after aggregation is done in the backend cloud.

#### 3.3.3. Autonomous Mobility Through SSL

**Figure 10:**
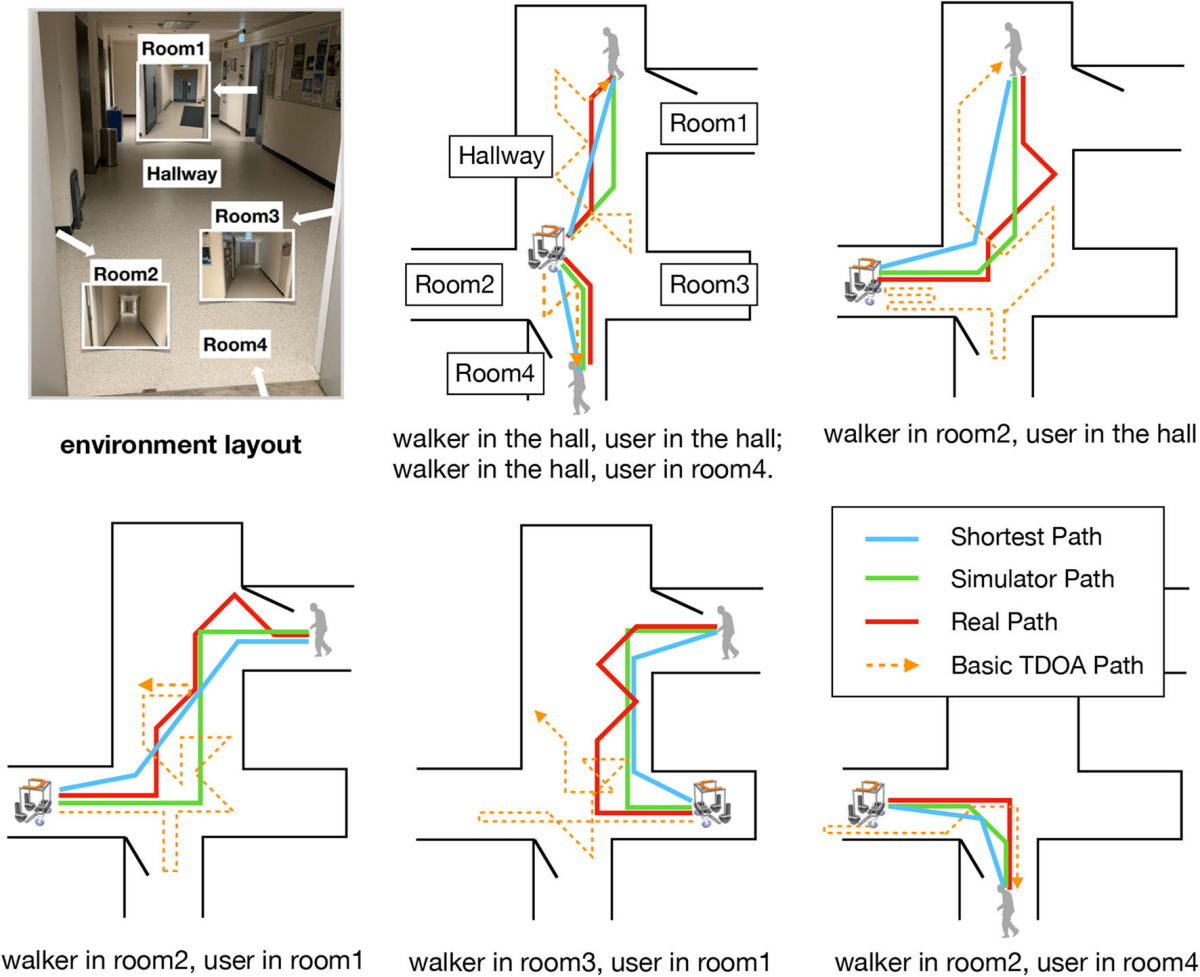
Walker routes in real-world tests, as compared to paths produced by basic TDOA method (geometric azimuth), simulator, and optimal shortest paths.

We experimented in a real-world home-like setting with one hall (maximum length over 10 m causing strong reverberation) and four separate regions (similar as rooms). The signal-to-noise ratio (SNR) can reach as low as 7 dB. Environment layout is given in Figure 10.

We first build the same environment in the GSound simulator. A microphone array with four microphones, whose maximum distance is 0.75 m, records sound data generated from one sound source. The length of each step of the walker is 1 m. By changing the positions of the walker and the sound source, we simulate in total 4,000 raw data samples (90% used as training dataset, 10% as test dataset) for training of the RL model. Figure 9C shows the training convergence curve, where a higher average reward per step indicates that the walker produces a better route toward the sound source.

The offline trained model is then deployed to the real-world scenario. In Figure 10, we compare the routes performed in reality to the routes derived by the simulator, as well as the optimal shortest routes computed. The difference between simulator paths and the shortest paths is mainly due to our defining eight discrete directions for the walker to move on. Our RL-based approach outperforms the basic geometric azimuth method (which directly uses calculated time-delay of each microphone pair to generate the basic TDOA path): the latter often makes the walker lost in the hall (due to high reverberation and the lack of reward mechanism), especially when the user summons the walker in different rooms. The “reality gap” (Tan et al., 2018) between real paths and simulator paths stems from the difference between the simulator and real-world environments: especially, for sound propagation in a multiple-room home with high reverberation, physical parameters of the real world are hard to be simulated exactly. Even so, our smart walker with RL model is able to automatically approach the user located in another room only based on voice signals. Our RL model is robust as it only needs offline training using data from the simulator and slight online tuning to achieve autonomous mobility in new environments.

## 4. Conclusion

This paper proposes a novel smart robotic walker platform to assist the elders with mild mobility impairment. We design a unique and sturdy mechanical structure that cooperates with sensors, and apply soft-robotic technology on the walker's handle to achieve better protection and richer sensing capabilities. A series of stability tests show that the walker has good resistance against external disturbance. The soft handle prototype meets our expectation and can provide useful information about the user with a low-cost solution.

We design a comprehensive finite-state machine model to detect user intention and emergency events in a timely manner through analyzing spatiotemporal pressure data collected from soft handle. We also develop a hands-free close-proximity front-following function through intelligent control using an IR sensor, a lidar, and NN-based gait classifier. A reinforcement learning-based sound source localization approach is implemented for summoning the walker to the user through voice signals. Field tests show that our walker can actively approach the user in a complex indoor environment through an acceptable path. All these intelligent functionalities achieved enables our walker to provide an elderly user with sufficient mobility safety and rich modes of human-robot interaction.

As future work, we will further investigate learning-based algorithms to learn more of user behavior through the soft handle interface. To make our front following more generic, we seek to collect data on more walking styles. For SSL, we plan to further reduce the “reality gap” when applying the RL model in the real world.

## Data Availability Statement

The raw data supporting the conclusions of this article will be made available by the authors, without undue reservation.

## Ethics Statement

Ethical review and approval was not required for the study on human participants in accordance with the local legislation and institutional requirements. The patients/participants provided their written informed consent to participate in this study. Written informed consent was obtained from the individual(s) for the publication of any potentially identifiable images or data included in this article.

## Author Contributions

XZ and ML designed software algorithms, performed the experiments, and wrote the corresponding part of the manuscript. ZZ designed hardware structure, performed the evaluations, and wrote the corresponding part of the manuscript. CZ analyzed user intention from data collected and wrote the corresponding part of the manuscript. YZ help design soft robotic sensors. JP, ZW, and CW conceived, supervised the project, and revised the manuscript. All authors contributed to the article and approved the submitted version.

## Conflict of Interest

The authors declare that the research was conducted in the absence of any commercial or financial relationships that could be construed as a potential conflict of interest.
